# Effect of Ozone Gas on Removal of Airborne Particles

**DOI:** 10.1055/s-0041-1741375

**Published:** 2022-04-18

**Authors:** Priscilla Alvarenga Agra, Patricia Alvarenga Agra, Marilia Fagury Videira Marceliano-Alves, Greice Maria Silva da Conceição, Sérgio Luiz de Lima Assumpção, Celso de Farias Crespo, Letícia Maria Borsarini Philippi, Renata Ximenes Lins

**Affiliations:** 1 Post Graduate Program in Dentistry, Health Institute of Nova Friburgo, Federal Fluminense University, Nova Friburgo, Rio de Janeiro, Brazil; 2 Immunobiological Technology Institute, Bio-Manguinhos, Oswaldo Cruz Foundation, Rio de Janeiro, Brazil; 3 Department of Endodontics and Dental Reseach, Iguaçu University, Nova Iguaçu, Brazil; 4 Research Department, Philozon Industry and Trade of Ozone Generator, Santa Catarina, Brazil

**Keywords:** ozone, ambiental sanitization, airborne particles, HEPA filter, clean rooms

## Abstract

**Objective** Airborne particles are one of the most important factors in the spread of infectious pathogens and must be monitored in healthcare facilities. Viable particles are living microorganisms, whereas non-viable particles do not contain microorganisms but act as transport for viable particles. The effectiveness of ozone in reducing these particles in a non-controlled room and a controlled cleanroom using high-efficiency particles air (HEPA) filter was analyzed in this study.

**Materials and Methods** Viable particles and non-viable particles sized 0.5 and 5 μm were quantified before and after ozonation in two different health environments: non-controlled (group 1) and controlled area, which was associated with a HEPA filtering system (group 2). Active air sampling using a MAS 100 was used to count the number of viable particles, while the number of non-viable particles/m ^3^ was obtained following the manufacturer's recommendations of the Lasair III 310C system.

**Results** Our results of the viable particles counting were not quantifiable and analyzed using statistical tests. Both groups showed a slight tendency to reduce the number of viable particles after ozonation of the environmental air. A statistically significant reduction of non-viable 5 μm particles after ozonation was observed in both groups (G1: *p* = 0,009; G2: *p* = 0,002). Reduction in the non-viable 0.5 μm particles after ozonation was observed only in group 2, associated with the HEPA filter. In group 1, after ozonation, a significant increase in 0.5 μm particles was observed, probably due to the breaking of 5 μm particles by ozone gas. Our results suggest that ozone gas can break 5 μm particles and, when associated with a HEPA filter, increases its effectiveness in removing 0.5 μm particles.

**Conclusion** Considering that 5 μm particles are important in the air transport of microorganisms, their reduction in the environment can be a relevant parameter in controlling the dissemination of infections.

## Introduction

The COVID-19 pandemic is a great concern and threat to the population worldwide and a huge challenge for healthcare professionals. In such times of major health crises, special attention is given to the importance of proper sanitization control of common environments, considering that air can be a vehicle for infectious agents. 1 Decontamination of the air of indoor health facilities is crucial in maintaining public health and requires continuous surveillance, improvement, and expansion of access. 2 3

Monitoring the air quality of indoor health facilities and removing airborne particles from such environments is recommended. 2 3 Control of environmental air can be performed by counting viable and non-viable particles. Non-viable particles are those that are unable to survive, grow, develop, and reproduce. Although airborne microorganisms are not free-floating or single cells, they often associate with 10 to 20 µm particles. 4

Particles classified as viable or non-viable can come from different sources, such as flaking of the skin in humans or due to movement when opening packages, for instance. 4 The method of counting viable particles has always been used to control the sterility of environmental air, as seen, for example in previous research. 5 They analyzed microbial contamination of the air in operating rooms of a Ghanaian hospital with an active air MAS-100 sampler. The authors verified the count of viable particles in 124 elective surgical procedures, both clean and contaminated. The study revealed an increased number of viable airborne particles during the procedures, exceeding the levels established by the current health legislation. 5

This methodology, although functional, has some disadvantages, such as cost, a longer time for reading the results, and biological risk of the operators who manipulate live microorganisms. With the emergence of modern portable particle counters, the daily count of non-viable particles is now being used in a variety of critical areas because it is simple and inexpensive. The implementation of this monitoring procedure allows for a real-time response to any deviation in the number of dispersed particles in the environment. 6

For these reasons, counting non-viable particles is a reliable diagnostic tool for measuring air quality. Furthermore, it presents a lower biological risk, is reproducible, and is cheaper than microbial counting. The main goal of monitoring non-viable particles is to demonstrate that a cleanroom is operating within the pre-established limits without directly evaluating the environment's sterility. It can, therefore, be used as a predictive parameter in monitoring contamination. The relationship of non-viable particles and viable airborne particles, concluding that both are indeed correlated, opens the possibility to use the non-viable particle count as an effective method of environmental monitoring. 6

Therefore, there is a tendency to apply the standards for non-viable particle counts of industrial cleanrooms to hospitals as well. These standards are based on the presence of particles sized 0.3 to 5 µm and are more practical than microbial sampling. Infectious pathogens, including fungi, bacteria, and viruses, can become airborne in moisture droplets of 5 to 10 µm after coughing or sneezing. Their small size allows them to remain airborne for several hours and be carried by drafts for considerable distances. 7

Currently, the most common laminar-flow system for air filtering and particle control is the high-efficiency particulate air (HEPA) filter, in which particles 0.3 µm or larger are removed with 99.97% efficiency. 8 The high efficiency of the HEPA filtering system depends on the complete absence of leaks, which leads to greater maintenance costs when compared with other types of filters. Additionally, the efficiency of a HEPA filter will increase as particles accumulate in the filter. However, this particle build-up will decrease the airflow rate, in turn, decreasing the number of air exchanges per hour that the unit can supply, requiring constant monitoring. 7

An alternative tool that has been studied as a potent sanitizing agent in the fields of medicine and dentistry, is ozone gas due to its already well-documented antimicrobial properties. 8 In addition, its therapeutic application has been the subject of numerous studies, not only for its antimicrobial properties but also for its anti-inflammatory action. It has been clinically used for wound healing, as a high O _2_ tension increases the formation of granulation tissue, favoring tissue repair. 9 In addition, it can promote the increased generation of growth factors and activate local anti-oxidant mechanisms; it also promotes tissue repair. 10 11

Ozone can inactivate microorganisms by damaging their genetic material. For these reasons, ozone has been widely used for the inactivation of microorganisms. 1 12 This gas is easily and cost-effectively produced and is a natural compound that quickly decomposes to oxygen with a half-life of ∼20 minutes. Also, it can effectively penetrate all parts of a room, including those of difficult access by conventional liquids or manual cleaning procedures. 13 Although ozone has proved effective for the *in vitro* inactivation of a series of microorganisms, including pathogenic bacteria and viruses of clinical relevance in nosocomial infections, its use in environmental and hospital disinfection or sterilization is still not widespread. 14 15

Even with vast literature underpinning the effectiveness of ozone gas on microorganisms regarding viable particles, so far only a few studies have evaluated its action on non-viable particles. Therefore, our experimental study proposes an investigation on the effect of ozonation on the number of viable and non-viable particles comparing a non-controlled and a controlled environment associated with a HEPA filter.

## Materials and Methods

### Environment Selection and Classification

Two different areas commonly found in health facilities were tested. In group 1, the non-critical, non-classified, or non-controlled area used in our tests was a 30 m ^3^ room used as an office. In group 2, the cleanroom selected was an N8 ISO 16444 grade D (Anvisa RDC n17/2010, 2010), 16 laboratory with 60 m ^3^ located at the Immunobiological Technology Institute, within Oswaldo Cruz Foundation.

### Ozonation Procedure

**Fig. 1:**
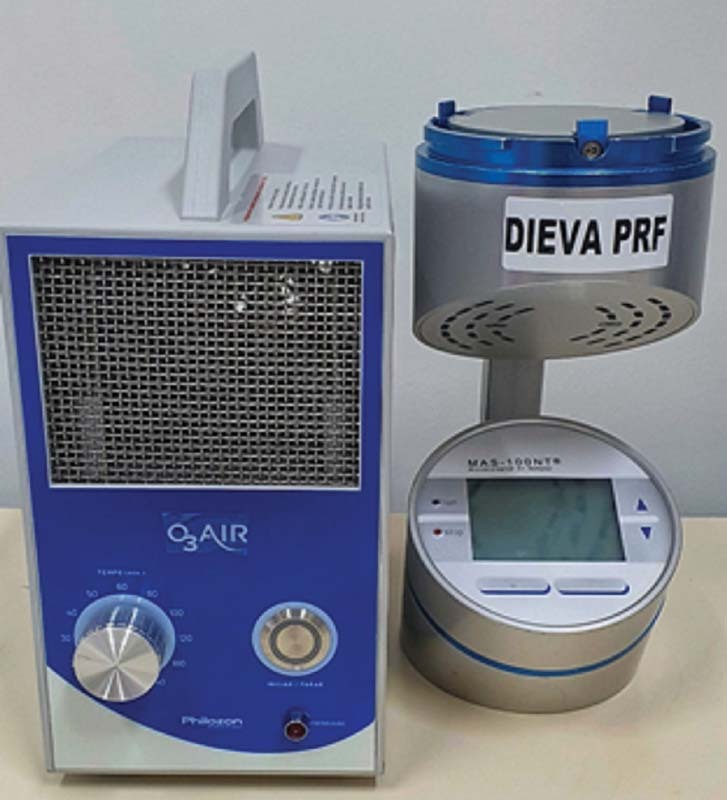
The O _3_ air ozone generator and microbial air monitoring system (MAS-100).

The ozone (O _3_ ) air ozone generator (Philozon, Balneário Camboriú, SC, Brazil) was used to disperse the ozone gas in the chosen environments ( Fig. 1 ). Using oxygen from environmental air, the generator produces 10 g/h of ozone at a flow rate of 1560 L/min. Following the manufacturer's instructions, to obtain an approximate concentration of 15 to 20 ppm volume (ppmv) of ozone in the environment, the application time was set by the volume of the treated area (30 minutes: 30 m ^3^ and 60 minutes: 60 m ^3^ ). Ozone output was previously confirmed by DN 800–03, a portable pump-suction ozone gas detector (Dino Purification Co., LTD, Guangzhou, China). All tests in each area were performed on three separate and consecutive days. Humidity and temperature were registered at the time of each test and considered in the final analysis of the results.

### Environmental Monitoring

For environmental monitoring in this experimental study, active methods of counting viable and non-viable particles were used in the selected environments before and after the ozonation procedure.

### Active Sampling of Viable Particles

Before and after ozonation, a microbial air monitoring system (MAS-100; Merck, Darmstadt, Germany) was used for 5 minutes at a flow rate of 100 L/min to count viable particles in the air ( Fig. 1 ). Two air-samplers were positioned in the tested room 60 and 120 cm from the ozone generator. The environmental air was aspirated through a perforated lid and impacted onto the surface of growth media (Tryptic Soy Agar) in a 90 mm Petri dish. After the air sampling procedure, the Petri dishes were incubated at 37°C for 48 hours. Colonies were counted and expressed as colony-forming units/cubic meters (CFU/m ^3^ ).

### Active Sampling of Non-Viable Particles

**Fig. 2:**
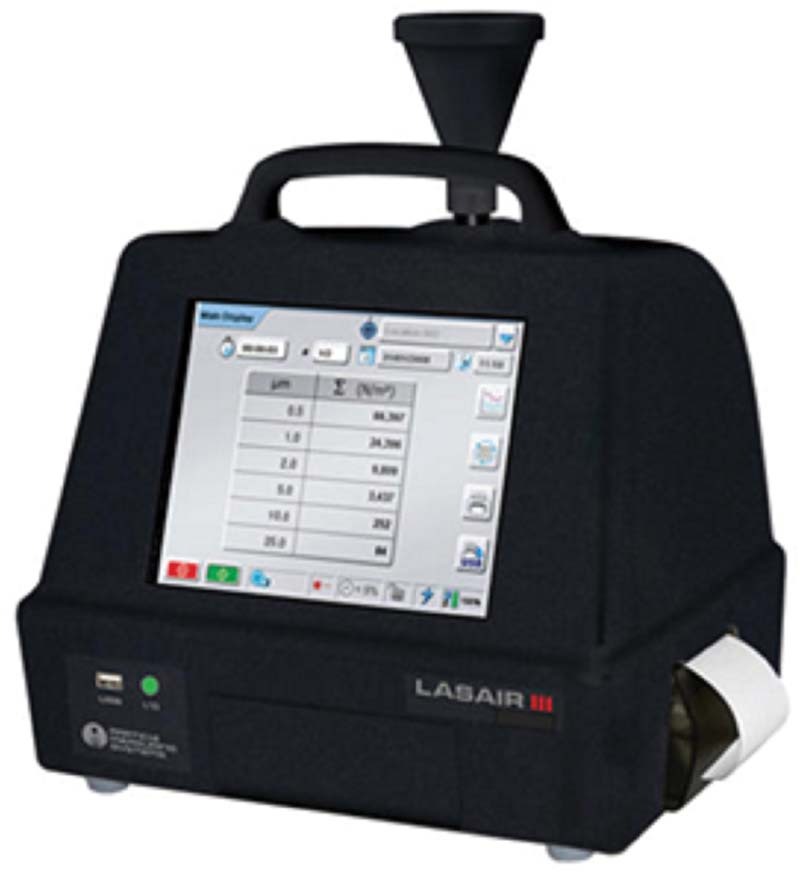
Lasair III 310C (Particle Measuring System, Colorado, EUA) particle counter.

Before and during the ozonation procedure, Lasair III 310C (Particle Measuring System, Colorado, EUA) particle counter was used to count non-viable particles by active sampling ( Fig. 2 ). The particle counter was positioned in the center of the tested environment. The sampling of non-viable particles followed the specific operating procedures and configurations of the particle counter. The 0.5 and 5 μm size particles were selected for counting according to the parameters indicated for N8 grade D cleanrooms described in ISO 16444 16 . The particle counter was adjusted to count the number of particles/m ^3^ and with a sampling volume of 0.283 m ^3^ (283 L).

### Statistical Analysis

The results were analyzed by the Mann—Whitney *U* nonparametric test using Statistica version 13.3 statistical program to assess the difference in medians before and after ozonation, considering *p* < 0.05 as significant.

## Results

### Environmental Monitoring

In all tests, the relative humidity (RH) and temperature were monitored. In group 2, grade D clean room, these parameters were 70% RH and 20°C. In group 1, non-classified area, temperature averaged at 22°C with 55% to 75% RH.

### Active Sampling of Viable Particles

In group 1, 12 samples were collected and in 5 of them, the microbial growth was such that it was not possible to count CFU for these samples. In three tests, it was possible to obtain a microbial count pre-ozonation and in 100% of these, a reduction in the number of colonies was observed after ozonation. In group 2, with the HEPA filtering system, it was possible to count the number of colonies in all samples and Table 1 shows a reduction trend in these results. Due to the impossibility of performing the CFU count in part of the trials, no statistical tests were performed, and the results were analyzed regarding only the percentage reduction.

**Table 1:** Viable particles counting (CFU/m ^3^ ) before and after ozonation

	Distance from O _3_ generator	Day test	UFC before O _3_	UFC after O _3_	Status
Group 1	1 m	Day 1	38	18	Reduction
		Day 2	Uncountable	Uncountable	Unchanged
		Day 3	Uncountable	134	Reduction
	2 m	Day 1	21	19	Reduction
		Day 2	163	96	Reduction
		Day 3	Uncountable	Uncountable	Unchanged
Group 2	1 m	Day 1	1	1	Unchanged
		Day 2	5	5	Unchanged
		Day 3	11	5	Reduction
	2 m	Day 1	0	0	Unchanged
		Day 2	3	0	Reduction
		Day 3	Uncountable	4	Reduction

### Active Sampling of Non-viable Particles

Table 2 depicts the results of the average, median, and standard deviation of group 1's particle counts after ozonation in each of the three days of trials. After the use of O _3_ in group 1, an 89% reduction in the median number of 5.0 μm particles was observed, while the median number of 0.5 μm particles increased 586%. Table 3 describes the average, median, and standard deviation of group 2's particle counts after ozonation in each of the three days of trials. After the use of O _3_ in group 2, a 97% and 100% reduction in the median number of particles was observed for particles sized 0.5 and 5 μm, respectively.

**Table 2:** Non-viable particles counting (particles/m ^3^ ) before and after ozonation in the Group 1

Particle size	Results before O _3_		Results after O _3_			Descriptive statistics	
		10 min	20 min	30 min	Average	RSD*	Median
Day 1							
0.5 μm/m ^3^	7.492.816	44.957.524	68.076.384	66.961.584	59.998.497	22%	66.961.584
5 μm/m ^3^	40.798	7.376	4.459	3.895	5.243	36%	4.459
Day 2							
0.5 μm/m ^3^	15.432.208	52.731.968	64.462.592	60.053.872	59.082.811	10%	60.053.872
5 μm/m ^3^	31.667	4.655	3.599	3.425	3.893	17%	3.599
Day 3							
0.5 μm/m ^3^	8.753.473	50.018.908	60.366.988	52.848.396	54.411.431	10%	52.848.396
5 μm/m ^3^	19.223	3.405	2.363	2.176	2.648	25%	2.363

**Table 3:** Non-viable particles counting (particles/m ^3^ ) before and after ozonation in the Group 2

Particle size	Baseline	Measurements after O _3_						Descriptive statistics		
		10 min	20 min	30 min	40 min	50 min	60 Min	Average	RSD*	Median
Day 1										
0.5 μm/m ^3^	1596	21	14	4	7	11	21	13	54%	13
5 μm/m ^3^	233	0	0	0	0	0	0	0	–	0
Day 2										
0.5 μm/m ^3^	1155	92	53	99	74	56	187	94	53%	83
5 μm/m ^3^	102	7	4	7	4	0	21	7	101%	6
Day 3										
0,5μm/m ^3^	1303	14	25	7	21	28	25	20	40%	23
5μm/m ^3^	141	0	0	4	0	0	0	1	245%	0

**Fig. 3:**
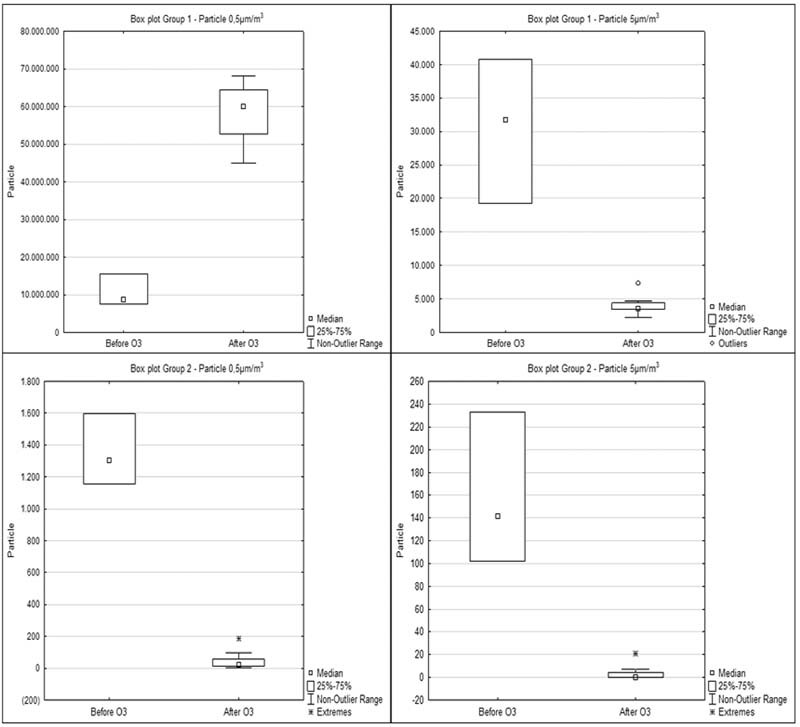
Box plot variation of non-viable particles counting (particles/m ^3^ ) before and after ozonation.

The results found in groups 1 and 2, before and after ozonation were compared using the Mann–Whitney *U* nonparametric test. In both groups, it was observed, with a 5% significance level, that there was a statistically significant difference ( *p* = 0.009 in group 1 and *p* = 0.002 in group 2) in the number of 0.5 and 5 μm particles before and after the use of O _3_ ( Fig. 3 ).

## Discussion

Airborne biological particles, including bacteria, fungi, and viruses, are commonly found in the breathing air. These microorganisms may serve as respiratory pathogens and if they are still viable after aerosolization and suspension in the air, they have the potential to cause respiratory diseases. Therefore, it is important to develop continued methods for controlling and improving environmental air quality. 1 17 18

Monitoring of air quality in healthcare facilities is mandatory. Textile fibers, dust particles, skin flakes, and respiratory aerosols, for example, can transport microorganisms and deposit them onto instruments and surfaces of surgical areas, leading to contamination and potential cross-infection. 5 6 A previous study sought to identify the generation and behavior of airborne particles due to the movement of the medical team when entering and exiting the operating room. The authors identified that airborne particles were generated from the floor and the shoes of the medical team. Particularly those sized 0.3 to 0.5 μm were carried up to the level of the operating table due to the movement of the team in the operating room. 5

Currently, the most used method for the reduction of particles in the environment is the HEPA filter. HEPA filters remove 99.97% of particles 0.3 μm or larger and create a homogeneous airflow in the operating room with little turbulence. However, they are expensive to install and maintain and demand continuous monitoring. 19 A previous work correlated airborne microbial contamination with the rate of air renewal in operating rooms. They concluded that microbial contamination decreased as the air renewal rate increased. 20 Only a few countries have established acceptable levels of microbial contamination for operating rooms with conventional ventilation and most recommend 20 air renewals per hour to get 50 ± 150 CFU/m ^3^ of air. 6 20

In 2018, a study characterized the type and concentration of bioaerosols in an operating room before and after sterilization and disinfection, finding growth of biological species even after the cleaning procedures. The authors reported that factors such as insufficient ventilation, inadequate HEPA filtering, high humidity, and a lack of proper post-surgical infectious residue management may have been the causes for the observed increase. These authors suggest designing better ventilation systems, replacing the HEPA filters, implementing more rigorous and frequent disinfection procedures, and controlling relative air humidity and room temperature. 21

Additionally, another work determined the factors that influence air contamination in a surgical medical center by active sampling methods. They found that even when the ventilation system was controlled with a HEPA filter, microbial concentration was far from the ideal levels during surgical activities. Inadequate air filtering was the main source of contamination. Additionally, the colony count significantly increased with the increment of 1°C in room temperature and was also correlated to the number of people in the room. 22

All these findings led to the search for additional sanitization tools and motivated us to investigate ozone in this study. Ozone can inactivate microorganisms by damaging their genetic material. For these reasons, ozone has been widely used for the inactivation of microorganisms. 1 12

Gaseous disinfectants were introduced in the market as an effective alternative for manual disinfection. 14 They have also been used as a co-adjuvant sanitization method leveraging the performance of HEPA filtering. Gaseous disinfectants as ozone have antimicrobial properties four times greater than liquid disinfectants, due to their uniform distribution and penetration. Beyond its effective diffusibility, ozone has a broad antimicrobial spectrum. When compared with chlorine, ozone gas requires a lower concentration and application time to effectively disinfect. 23

In a recent study, the authors evaluated the microbial decontamination after an ozone application within a triage area that receives patients with suspected cases of COVID-19. Air and surface samples were collected to evaluate the efficiency of ozone for microbial reduction. The authors concluded that an ozone system within the rooms of a COVID-19 triage area was highly efficient and led to significant reductions ( *p* < 0.05) in bacterial and fungal counts. Furthermore, it is possible to assume that ozone application could also be useful for inactivating the SARS-CoV-2 virus 23

Hudson et al, in 2008, develop a practical method to test the anti-viral properties of ozone in a mobile apparatus that could be used to decontaminate rooms in health care facilities, hotels, and other buildings. Maximum antiviral efficacy was obtained after a short period of time in a high humidity (>90% RH) and gas concentration 20 to 25 ppm. All 12 viruses tested, on different hard and porous surfaces, and in the presence of biological fluids, could be inactivated by at least 3 log10. 24

Another study analyzed the influence of different species of microorganisms, relative humidity, and ozone dose on the disinfection of surfaces using this gas. The authors concluded that the survival of microorganisms and the dose of ozone (ozone concentration times the exposure time) have an exponential relationship. Furthermore, they found that ozone was a more effective germicide when RH increased. This might be related to an increase in free radicals generated from ozone's reaction with water vapor from more humid environments. Finally, the authors concluded that ozone is highly effective and is a reliable treatment for contaminated surfaces. 12

Some factors such as temperature, RH, and turbulence are decisive factors in the dispersion and dissemination of infectious agents in indoor environments. Therefore, from a methodological point of view, field studies should try to control these potential variables as well as include effective ventilation systems. Considering these parameters, our study included ozonation of a controlled and non-controlled environment, both free of human activity around the equipment to avoid any air turbulence. Additionally, the temperature and RH of both the controlled and non-controlled rooms were maintained at ∼20°C and 60% RH throughout the experiment. 2

The effectiveness of ozone (aqueous and gaseous) as an alternative sanitization technology to conventional disinfectants in the reduction of microbial contamination of water and air was already assessed. The authors found that the most effective reduction in microbial load occurred after 20 minutes of ozonation. They concluded that ozone treatments were effective against microbial contaminants, reducing the CFU of the investigated microorganisms, making ozone an extremely promising alternative. Furthermore, the authors highlighted that due to the room's large volume (85 m ^3^ ), there was an elevated dispersion of ozone, recommending that the experiment be repeated in a smaller environment to evaluate the positive impact of ozone on airborne microorganisms. 14

Taking into account the authors' suggestion, in our study, ozone was applied in smaller rooms to minimize gas dispersion. The non-controlled area had footage of 30 m ^3^ and the cleanroom of 60 m ^3^ , being ozonized respectively for 30 and 60 minutes, thus obtaining an average of 10 minutes per 10 m ^3^ , as recommended by the manufacturer Philozon. Although our results suggested a trend in reducing the viable particle count (CFU/m ^3^ ), this is just an initial observation, further tests must be performed on the number of viable particles, which is one of the limitations present in this study.

However, we were able to quantitatively analyze non-viable particles, which is the main recommended method for monitoring clean rooms (ISO 16444: 2015). 16 In previous work, methods to monitor and interpret airborne particles in a cleanroom were compared. The authors concluded that viable and non-viable particles are indeed correlated, recommending the count of non-viable particles as a routine procedure in preventing an increase in microbial air contamination. 6 Besides reducing the contamination risk of those manipulating live microorganisms, this method can also be used as a diagnostic tool for air treatment. Counting viable particles, even if by an automated process, requires expensive culture media and longer incubation and interpretation times. 1 4 20

In our study, we showed that ozone gas reacts with non-viable particles significantly reducing the number of 5 μm particles in both tested rooms, non-controlled and controlled with a HEPA filtering system. Furthermore, our results showed that in the non-controlled environment, without the HEPA filter, ozone reacted with the particles therein promoting a substantial decrease in 5 μm particles. However, due to their fragmentation, a significant increase in the number of 0.5 μm particles was observed 25 26 ( Fig. 3 ).

These results are in accordance with a previous one that also found the increased formation of particles after indoor ozonation in a non-controlled room. The authors explored the effects of ozonation in the presence of a common terpene source in residential dwellings. The results of their study suggest that the occupants of residential dwellings may also be exposed to elevated levels of fine and ultra-fine particles when an ozone generator is employed in a residential setting, particularly during periods of relatively high terpene concentrations, e.g., during the use of pine oil-based cleaners or scented deodorizers, what should be a concern with respect to elevated inhalation exposure. 26

Ozone's reaction with several compounds occurs in two different ways and both coexist. One involves direct reactions of molecular ozone and the other occurs through reactions of its subproducts, such as free radicals. The reactions between ozone and some unsaturated hydrocarbons can be an important source of secondary pollutants, including free radicals, carbonyl, and carboxylic acids. The formation and effect of these secondary organic particles in human health need to be clarified. 12 26

In contrast, in the controlled room, this increase in 0.5 μm particles was not observed as these particles were almost eliminated when ozone was applied in a room with a HEPA filtering system. These data suggest that the particles cleaved by ozone were effectively filtered from the environment by the HEPA filter because a significant reduction in the total particle count (0.5 μm and 5 μm) was observed when compared with the counts obtained before ozonation, just with the HEPA filter. It is clear from our tests that ozonation improved HEPA filtering. 22 26

The disadvantage of indoor ozonation is ozone's toxicity to the pulmonary alveoli when inhaled at high concentrations. For this reason, it cannot be used in constantly populated areas. This means that ozone can only be used in environments that can be closed off and emptied during application. Although toxic at high concentrations, ozone quickly dissociates into oxygen and, if necessary, a catalytic converter can be used to reverse ozone to oxygen. The protocol used in this study followed the manufacturer's instructions which suggest that in a 30 m ^3^ room, the ozone generator should remain active for 30 minutes in a room free of people. Therefore, although this is indeed a limiting factor, it does not impede the use of ozone. 24

## Conclusions

In this study's conditions, ozone gas was able to significantly reduce the amount of 5 μm particles regardless of the environment's classification. Also, the HEPA filter increased the effectiveness in the reduction of 0.5 μm particles. As a limitation of our study, the results of the active sampling of viable particles could not be statistically analyzed and were excluded from our conclusion. Considering that particles 5 μm or larger are important in the air transport of microorganisms, their reduction in the environment is an important factor in controlling cross infections. Ozone gas has proven to be an effective tool in controlling such infections and can be applied to a variety of indoor environments, those controlled with a HEPA filter or common areas such as offices, homes, and hotels. Further investigations into the formation of secondary organic particles must be conducted.

## References

[1] Charise D B Vieira K P Rossato L G Microbiological environmental monitoring after the use of air purifier ozone generator Ozone Sci Eng 2012 34 03 225 230

[2] Ijaz M K Zargar B Wright K E Rubino J R Sattar S A Generic aspects of the airborne spread of human pathogens indoors and emerging air decontamination technologies Am J Infect Control 2016 44(9, Suppl):S109 S120 27590695 10.1016/j.ajic.2016.06.008 PMC7115269

[3] Alimohammadi M Naderi M Effectiveness of ozone gas on airborne virus inactivation in enclosed spaces: a review study Ozone Sci Eng 2021 43 01 21 31

[4] FDA, Guidance for Industry: Stehle Drug Products Produced by Aseptic Processing-Current Good Manufact Uring Practice, 2004

[5] Sunagawa S Koseki H Noguchi C Airborne particle dispersion around the feet of surgical staff while walking in and out of a bio-clean operating theatre J Hosp Infect 2020 106 02 318 324 32702464 10.1016/j.jhin.2020.07.016

[6] Stauning M T Bediako-Bowan A Andersen L P Traffic flow and microbial air contamination in operating rooms at a major teaching hospital in Ghana J Hosp Infect 2018 99 03 263 270 29253624 10.1016/j.jhin.2017.12.010

[7] Raval J S Koch E Donnenberg A D Real-time monitoring of non-viable airborne particles correlates with airborne colonies and represents an acceptable surrogate for daily assessment of cell-processing cleanroom performance Cytotherapy 2012 14 09 1144 1150 22746538 10.3109/14653249.2012.698728 PMC4165074

[8] Medical Advisory Secretariat Air cleaning technologies: an evidence-based analysis Ont Health Technol Assess Ser 2005 5 17 1 52 PMC3382390 23074468

[9] Ahmedi J Ahmedi E Sejfija O Agani Z Hamiti V Efficiency of gaseous ozone in reducing the development of dry socket following surgical third molar extraction Eur J Dent 2016 10 03 381 385 27403058 10.4103/1305-7456.184168 PMC4926593

[10] Monteiro C GJ Vieira E M Emerick C Ozonated oil effect for prevention of medication-related osteonecrosis of the jaw (MRONJ) in rats undergoing zoledronic acid therapy Clin Oral Investig 2021 25 12 6653 6659 10.1007/s00784-021-03951-3 33895916

[11] Faccini M Agostini F Drieu T Preliminary histological evaluation of the application of ozone in the first days of orthodontic force induction in animal model Eur J Dent 2022 16 01 122 129 34428846 10.1055/s-0041-1731886 PMC8890936

[12] Li C S Wang Y C Surface germicidal effects of ozone for microorganisms AIHA J (Fairfax, Va) 2003 64 04 533 537 10.1202/559.1 12908871

[13] Dharan S Pittet D Environmental controls in operating theatres J Hosp Infect 2002 51 02 79 84 12090793 10.1053/jhin.2002.1217

[14] Martinelli M Giovannangeli F Rotunno S Trombetta C M Montomoli E Water and air ozone treatment as an alternative sanitizing technology J Prev Med Hyg 2017 58 01 E48 E52 28515631 PMC5432778

[15] Jezini C Gama M S Moura B F In vitro antimicrobial activity of ozonated sunflower oil against antibiotic-resistant *Enterococcus faecalis* isolated from endodontic infection Ozone Sci Eng 2020 43 04 378 383

[16] ISO 14644–1 Cleanrooms and associated controlled environmentsPart 1: Classification of air cleanliness by particle concentration 2015

[17] Hallier C Williams D W Potts A J Lewis M A A pilot study of bioaerosol reduction using an air cleaning system during dental procedures Br Dent J 2010 209 08 E14 20953167 10.1038/sj.bdj.2010.975 PMC7091833

[18] Guo Z D Wang Z Y Zhang S F Aerosol and surface distribution of severe acute respiratory syndrome coronavirus 2 in hospital wards, Wuhan, China, 2020 Emerg Infect Dis 2020 26 07 1583 1591 32275497 10.3201/eid2607.200885 PMC7323510

[19] McHugh S M Hill A D Humphreys H Laminar airflow and the prevention of surgical site infection. More harm than good? Surgeon 2015 13 01 52 58 25453272 10.1016/j.surge.2014.10.003

[20] Vonci N De Marco M F Grasso A Spataro G Cevenini G Messina G Association between air changes and airborne microbial contamination in operating rooms J Infect Public Health 2019 12 06 827 830 31155407 10.1016/j.jiph.2019.05.010

[21] Dehghani M Sorooshian A Nazmara S Baghani A N Delikhoon M Concentration and type of bioaerosols before and after conventional disinfection and sterilization procedures inside hospital operating rooms Ecotoxicol Environ Saf 2018 164 277 282 30121503 10.1016/j.ecoenv.2018.08.034 PMC6151147

[22] Fu Shaw L Chen I H Chen C S Factors influencing microbial colonies in the air of operating rooms BMC Infect Dis 2018 18 01 4 29291707 10.1186/s12879-017-2928-1 PMC5749012

[23] Hilária M Geovana Di Thauan G Ozone application in COVID-19 triage areas and its efficiency of microbial decontamination Ozone Sci Eng 2021 43 04 306 316

[24] Hudson J B Sharma M Vimalanathan S Development of a practical method for using ozone gas as a virus decontaminating agent Ozone Sci Eng 2009 31 03 216 223

[25] EudraLex - The Rules Governing Medicinal Products in the European UnionVolume 4 - Good Manufacturing Practice Medicinal Products for Human and Veterinary Use, 2010. Accessed on June 2021 at:https://ec.europa.eu/health/system/files/2016-11/2009_06_annex13_0.pdf

[26] Hubbard H F Coleman B K Sarwar G Corsi R L Effects of an ozone-generating air purifier on indoor secondary particles in three residential dwellings Indoor Air 2005 15 06 432 444 16268833 10.1111/j.1600-0668.2005.00388.x

